# Steered Pull Simulation to Determine Nanomechanical Properties of Cellulose Nanofiber

**DOI:** 10.3390/ma13030710

**Published:** 2020-02-05

**Authors:** Ruth M. Muthoka, Hyun Chan Kim, Jung Woong Kim, Lindong Zhai, Pooja S. Panicker, Jaehwan Kim

**Affiliations:** Creative Research Center for Nanocellulose Future Composites, Department of Mechanical Engineering, Inha University, 100 Inha-ro, Michuhol-ku, Incheon 22212, Korea; mwongelinruth@gmail.com (R.M.M.); Kim_HyunChan@naver.com (H.C.K.); jw6294@naver.com (J.W.K.); duicaofei@naver.com (L.Z.); pooja.panicker7@gmail.com (P.S.P.)

**Keywords:** cellulose nanofiber, molecular dynamics simulation, hydrogen bond, mechanical properties, pull simulation

## Abstract

Cellulose nanofiber (CNF) exhibits excellent mechanical properties, which has been extensively proven through experimental techniques. However, understanding the mechanisms and the inherent structural behavior of cellulose is important in its vastly growing research areas of applications. This study focuses on taking a look into what happens to the atomic molecular interactions of CNF, mainly hydrogen bond, in the presence of external force. This paper investigates the hydrogen bond disparity within CNF structure. To achieve this, molecular dynamics simulations of cellulose Iβ nanofibers are carried out in equilibrated conditions in water using GROMACS software in conjunction with OPLS-AA force field. It is noted that the hydrogen bonds within the CNF are disrupted when a pulling force is applied. The simulated Young’s modulus of CNF is found to be 161 GPa. A simulated shear within the cellulose chains presents a trend with more hydrogen bond disruptions at higher forces.

## 1. Introduction

Since the discovery of cellulose, it has undergone extensive research from different research groups with the aim of understanding the reasons behind its excellent physical and chemical properties. The quest to explore cellulose for many applications still continue with many successes in unveiling the ‘cellulose mysteries.’ Due to the bioavailability, biodegradability, renewability, and above all the outstanding mechanical properties of nanocellulose, it is a cost-effective building block owing to its extensive range of applications in nearly all disciplines. The subject has raised intensive interest in cellulose nanofiber (CNF) mainly due to its ability to make strong bond networks [1,2]. Cellulose degree of polymerization can be placed anywhere from several glucose units to approximately 20,000 [3]. The monomer rings are connected to each other by a β(1-4) glycosidic linkage. So, to say, this simply means that the oxygen atom bound to the C (1) of one glucose ring covalently bonds the carbon atom C (4) of the adjacent glucose ring [4]. Cellulose nanofiber consists of several chains (30–40) where each chain has a chemically reducing end and a non-reducing end. 

There are three main types of hydrogen bonds (H-bonds) within the cellulose network; namely intra-molecular hydrogen bonds, inter-molecular hydrogen bonds, and intra-sheet hydrogen bonds. The inherent intra-molecular hydrogen bonds significantly contribute to the excellent physical properties of the CNF structure and include the O(2)H–O(6) H-bond and the O(3)H–O(5) H-bond, where in both cases an OH group from one unit bonds to the O atom of the next unit within the same chain [5]. Regarding donor-acceptor H-bond analogy, the donor atom is the oxygen atom covalently bonded to a hydrogen atom, O(i)H, which forms a secondary interaction with the acceptor atom of the next unit having lone pair electrons. Inter-molecular hydrogen bonds exist between atoms in the adjacent chains. In the case of cellulose, the donor O(6)H group forms H-bond with the O(3) atom as well as the O(2) atom of the immediate next chain [6]. Lastly, cellulose structure capably forms H-bonds within the cellulose sheets as depicted in the sheets arrangement of the CNF. The overall H-bond networks within the cellulose chains, between the chains and within the sheets partially contributes to the overall strength depicted throughout the cellulose nanofibers. It has been a journey with researchers finding interest in understanding these mechanisms at atomic level. 

Cellulose research has been advanced by many analytical techniques. Computational chemistry is an advanced analytical technique that has gradually developed since the 1980s. Molecular dynamics (MD) is a framework for carrying out relevant simulations of cellulose [7]. Various force-fields have been explored for accurately modelling CNF structure [4,5,6,8,9,10,11]. An improved force field OPLS-AA (All-Atom Optimized Potential for Liquid Simulations) extended for well definition of carbohydrates was selected for this study. An investigation of desorption processes of cellulose in water was carried out by potential of mean force simulations using CHARMM (Chemistry HARvard Macromolecular Mechanics) force field [9]. Cellulose fibers consist of crystalline regions and amorphous regions. Elastic modulus of the crystalline regions of cellulose is quite important while aiming for its use in composites and structural applications. The elastic properties of the crystalline regions of cellulose have been studied by theoretical approaches or experimentally using X-ray diffraction, Raman spectroscopy, wave propagation, and AFM (Atomic Force Microscopy) since mid-1930. In 1995, structures and Young’s moduli of cellulose I and II were calculated on molecular dynamics basis with values of Young’s modulus for cellulose I reported to be 134–135 GPa [12]. Molecular dynamics modeling of regenerated cellulose fibers was used to investigate its mechanical properties [13]. At room temperature, the longitudinal modulus of regenerated cellulose was found to be 155 GPa. Elastic modulus of cellulose Iβ crystalline region was calculated by molecular dynamics simulation [14] which reported Young’s modulus values ranging from 124–155 GPa. Compressive and tensile deformation of various models of structures of crystalline regions of cellulose Iα, Iβ and cellulose II were studied by molecular mechanics modeling [15]. They reported chain stiffness of cellulose Iα as 136–155 GPa, 116–149 GPa for cellulose  Iβ, and 109–166 GPa for cellulose II. Thermal response of cellulose Iβ structure and its influence in cellulose’s properties were studied where a longitudinal Young’s modulus of 156 GPa was reported at 300 K and 117 GPa at 500 K [16]. An atomistic modeling study to investigate the mechanical properties of single cellulose chains was conducted [17] and reported axial modulus predictions of between 109.4 GPa for large strain and 254.8 GPa for small strain. Mechanical properties of cellulose nanofibers investigated by atomistic molecular dynamics simulation reported an elastic modulus of 157 GPa for crystal cellulose [18]. A Young’s modulus of around 120 GPa was reported from molecular deformation of the coarse grain model of cellulose nanocrystals [19]. Atomistic simulations of uniform deformation and nanoscale indentation methods were used to study the elastic modulus and hardness of cellulose Iβ crystal [20]. Elastic moduli of 139.5, 7, and 28.8 GPa were reported for the *c, a*, and *b* directions, respectively. The same research team reported an axial modulus of 107.8 GPa and transverse moduli of 21.6 and 7.6 GPa in the x- and y- directions, respectively [21]. Elastic Young’s modulus of various cellulose allomorphs was reported to be 138, 112, and 101 GPa for cellulose Iβ, II, and III*_I_*, respectively [5].

In this paper, a steered MD simulation is performed to investigate the nanomechanical properties of cellulose nanofiber. Steered MD simulation basically entails applying an external force to a group of atoms, in which case, one can opt to keep other groups of atoms fixed, where various behaviors of interest are then investigated. In a pull simulation case, we take into account cellulose nanofiber structure in order to investigate the mechanisms beyond its mechanical properties. We were principally interested in understanding in depth the molecular behavior in the structural aspect of CNF, especially the secondary molecular interactions, the H-bond network. The significance of this research is to give a better insight and to elucidate the molecular structure behavior that can be exploited to enhance compatibility of CNF and other polymers by focusing on the crucial H-bond network to form composites that can possibly be much stronger than the CNF itself.

## 2. Computational Methodology 

### 2.1. Simulation Model of Cellulose Nanofiber (CNF)

The molecular model of the cellulose Iβ was modelled based on X-ray diffraction measurements and unit cell specification [22]. A hexagonal CNF model was made with 36 chains using cellulose builder [23,24]. This model was then used as a basis to produce 18 chain model forming six sheets [25,26,27]. Each chain consisted of 20 glucose residues, and a total of 7614 atoms.

### 2.2. Simulation Description

The atomistic molecular dynamics simulations are carried out using the GROMACS (GROningen Machine for Chemical Simulations) software (Stockholm Center for Biomembrane Research, Stockholm, Sweden) version 5.1.2 [28]. To parameterize the cellulose molecules, we used the all-atom optimized potentials for liquid simulations (OPLS-AA) force field extended for carbohydrate simulations [29,30,31,32,33]. Periodic boundaries were applied in all directions to provide a viable numerical means of strain application without necessarily imposing artificial constraint on the chains themselves, and for ease to isolate material response from that caused from crystal surfaces [17]. 

The cellulose nanofibers structure is placed in the center of the simulation box with the axis of the CNF oriented along the z-direction. For solvation, water molecules are added to the simulation box. We applied transferable intermolecular potential with 3 points (TIP3P) water model compatible with the chosen force field, OPLS-AA, and Cl^-^ and Na^+^ ions added to keep the system neutral. This water model ensures that the CNF is suitably packed in all the Cartesian x, y, and z directions by at least 1 nm [10,11,34]. The bonds within the cellulose chains are described by quadratic potentials and the hydrogen atoms are dynamic, hence allowed to form spontaneous transient H-bond networks. The simulation temperature was set at 300 K. The system consisted of 7614 CNF atoms, 333,564 water molecules, 210 chlorine ions, and 210 sodium ions. Prior to the actual MD simulations, energy minimization and equilibration steps were carried out. For the energy minimization, the MD simulation software (Stockholm Center for Biomembrane Research, Stockholm, Sweden), GROMACS in this case, moves all atoms around the simulation box to locate the lowest local energy state for all the atoms under simulation. This ensures that the atoms are reasonably distanced from each other at the beginning of the simulation. The system is energy minimized using the steepest descent algorithm to let the simulation system reach a state of its lowest energy. Equilibration, on the other hand, involves a simulation that is run without any external force(s) to attain a stable configuration. Two equilibrations are carried out in the constant temperature and volume ensemble (NVT) as well as constant temperature and pressure ensemble (NPT). The final MD production is performed for 200 ps at 300 K using the Berendsen thermostat and the simulation pressure is maintained at 1 atm using the Berendsen barostat. Atomic coordinates are saved for every 500 steps. The Velocity-Verlet algorithm is employed for integration.

The simulations for investigating mechanical properties are conducted using the pull code (steered molecular dynamics) that is integrated in GROMACS. Two groups are selected in the fiber and a constant force is applied between centers of mass of the two groups. This study focuses on two sets of different pulling mechanisms as described below.

GROMACS MD simulation program allows the exploitation of user-defined groups consisting of atoms as per user’s preferences. In our case, we made use of freeze group. According to the GROMACS program, atoms of the frozen group are kept stationary throughout in the subjected dynamics and therefore, the said atoms cannot be moved by constraints. We employed this scheme to create a fixed end at one end of our cellulose nanofibers and carried several pull simulations as discussed below in Cases A and B.

**Figure 1:**
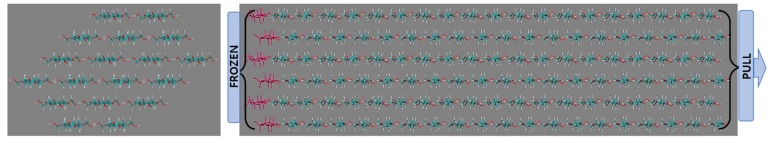
Case A: Representation of cellulose nanofiber (CNF) structure orientation with clear indication of frozen atom groups and pulled group of CNF simulation structure as indicated by the naming arrows in the diagram.

Case A: Atoms on one end of the CNF (front of the fiber) were fixed by freezing the first residue of each chain while a constant pulling force is applied to the other group (back end of the fiber) as illustrated in Figure 1. A series of individual pull simulations were performed at a temperature 300 K at different pulling forces: 1000, 2500, 5000, 7500, 10,000, 15,000, 20,000, and an extreme force, 50,000 kJ/mol/nm for 200 ps.

**Figure 2:**
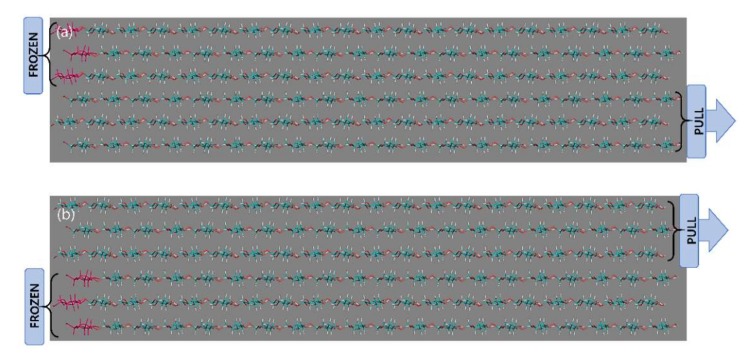
Case B for (**a**) freeze upper (front) pull bottom (back) denoted by FUPB, and (**b**) freeze bottom (front) pull upper (end) denoted by FBPU throughout the paper.

Case B: Different combinations of freeze and pull groups are explored. This is to investigate separation mechanisms and corresponding outcome between CNF chains. Only 5000, 10,000, and 15,000 kJ/mol/nm pulling forces are applied at 300 K for 200 ps. Based on the former pull combination described in (i), the frozen and the pull groups are varied to give different combinations as shown in Figure 2. 

In Case B, only the bottom half of the front end is frozen while the pulling force is applied to the upper half portion on the back end and vice versa i.e., upper half of front end is frozen while the bottom half of back end is pulled, denoted as FUPB where then, the situation is interchanged where lower half of front end is frozen and upper half pulled, denoted as FBPU. 

## 3. Results and Discussion

### 3.1. CNF Structure after Simulation

**Figure 3:**
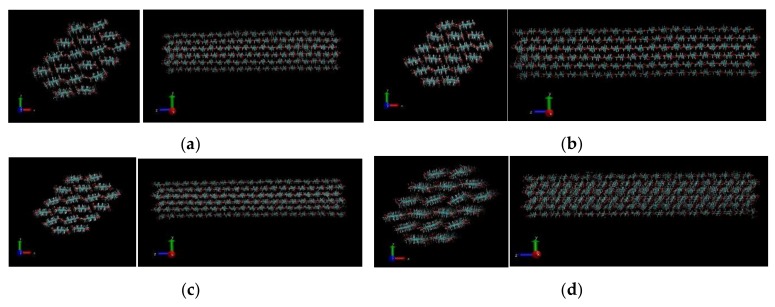
Case A: Structures of CNF after pull simulation at 200 ps for: (**a**) 1000 kJ/mol/nm, (**b**) 5000 kJ/mol/nm, (**c**) 20,000 kJ/mol/nm, and (**d**) 50,000 kJ/mol/nm.

In the pull simulation for Case A, the observed structures at the end of the simulations are presented in Figure 3. Only a representation of the low pulling force and high pulling force is provided. The integrity of the connecting chains of CNF is observed to be more compact at the smallest applied force. However, as the pulling force increases sufficiently, a structure with revealing disruption in bonding is observed. This phenomenon can be ascribed to the opening up of hydrogen bonding network. Under applied force, the hydrogen bonds in the CNF are susceptible to disruption as stretching of the bonds leaves the chains vulnerable to possible sliding with respect to one another. As shown in Figure 3 below, at low pulling force (1000 kJ/mol/nm), the disruption of the molecular structure of CNF is minimal and observed to increase as the pulling force increases with an extreme disruption depicted from Visual Molecular Dynamics (VMD) at extreme pulling force of 50,000 kJ/mol/nm. The reasons behind the structural changes associated with the applied pulling force are explained later.

**Figure 4:**
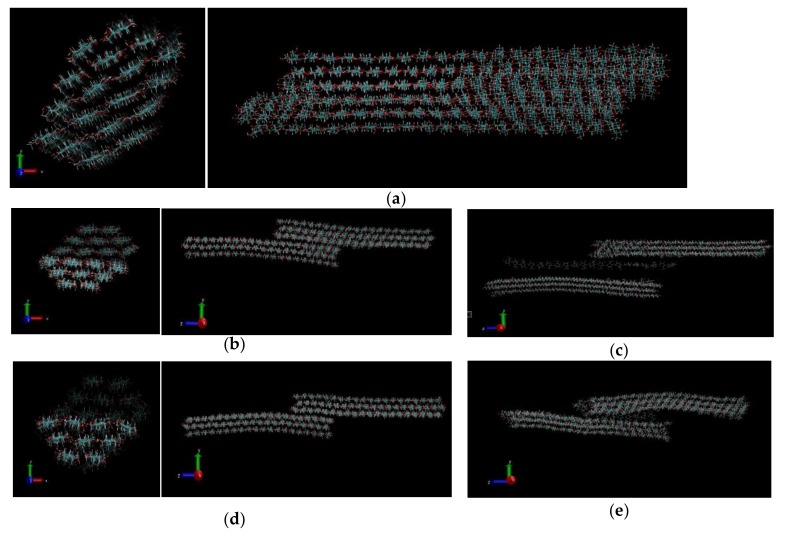
Case B: Structures of CNF after half pull simulations at: (**a**) 5000 kJ/mol/nm at 200 ps, (**b**,**c**) 10,000 kJ/mol/nm at 129 ps and 200 ps, respectively, and (**d**,**e**) at 50,000 kJ/mol/nm at 97 ps and 200 ps, respectively.

For pull scenario in case B, the resulting structure after simulation is represented for three pull forces 5000, 10,000, and 50,000 kJ/mol/nm as shown in Figure 4. We modeled the simulation as described in Case B of the pulling mechanism, and the representation of the molecular structure at the end of each simulation observed under Visual Molecular Dynamics software as represented in Figure 4 as snapshots of the VMD movie. At a low pulling force (5000 kJ/mol/nm), the CNF chains are observed to slowly separate along the interface of the fixed chains and the chains subjected to the pulling force. At low pull force, there was no observed separation of the chains for the greater part of the simulation time. However, towards the end of the simulation time, the chains were observed to separate slowly to the extent presented in Figure 4a. The sliding mechanism of cellulose nanofibers can be attributed to successive sliding of CNF atoms on top of each other. At 10,000 kJ/mol/nm and 50,000 kJ/mol/nm, separation was observed within the first few picoseconds of the MD simulation. Complete separation was observed at the end of the simulation at these higher pull forces. In addition, it was noted that complete separation at 50,000 kJ/mol/nm in Figure 4d,e was attained faster (within few picoseconds of simulation) than that observed at 10,000 kJ/mol/nm in Figure 4b, c. The overall number of hydrogen bonds after the simulations were also calculated.

### 3.2. Strain Response

**Figure 5:**
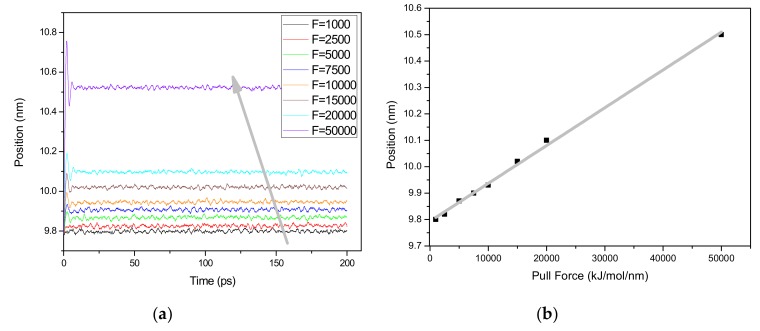
Strain response over simulation time: (**a**) With time and (**b**) with pulling force.

In basic mechanics of Hooke’s law, the extent of stretching produced by a material in response to the applied pulling force (stress) defines strain. In this study, pull forces are applied with different levels for each simulation. All simulations are carried out under the similar conditions; equilibration and energy minimization. Figure 5 shows the strain response of Case A under varying pulling force. All individual simulations are done from the original position and the final position is recorded at the end of the simulation. Strain is measured in relation to length. From a molecular structure level, when a pulling force is applied to a material, the pulling force is experienced by the atoms in the lattice structure of the material. The introduction of the pulling force displaces some atoms of the material away from the others. The position of the CNF after being subjected to different pulling forces is used to provide a general view of the possible displacement of the CNF atoms. Strain can be attributed to displacement which translates to deformation of a material’s molecular structure. Figure 5a shows the position of CNF with time during the entire simulation at different pulling forces. The degree of extension of CNF with respect to the applied force from the original position to the final position is directly related to the level of pulling force applied. At lower pull force, the extension or strain experienced by the CNF molecular structure is lower compared to that observed at higher pulling force cases. Figure 5b shows the position of CNF with pulling force as averaged from Figure 5a. Treating CNF as an elastic material, we could arguably agree that any applied force to the CNF produces a considerable amount of displacement, herein represented in terms of position. At different pulling forces of the individual simulations, the effective displacement is recorded as the final position from the original position as indicated in Figure 5b. A significantly large strain is observed immediately after the force is applied and settled to a constant value. This behavior is also clearly observed in VMD movie of the simulation and is consistent to observations of other research groups [18]. Under applied stress, the CNF chains starts to open up and stretch from the relaxed state of the CNF before the pull force is applied. If the pull force is not withdrawn, the bonds within the cellulose structure could begin to slowly stretch depending on the magnitude of the applied pull force. Our findings agree closely to the previous report [18] where a sudden increase in strain upon application of pulling force has been reported and a slow increment of the strain thereafter. 

### 3.3. Disparity of Hydrogen Bonds

**Figure 6:**
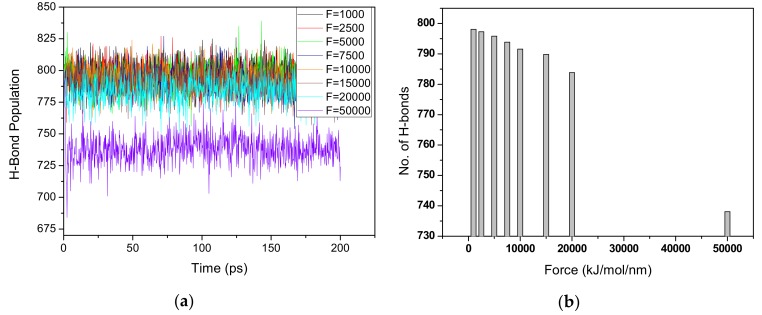
Hydrogen disparity after pull simulation of Case A: (**a**) H-bonds profiles and (**b**) averaged H-bonds.

In simple terms, a hydrogen bond in a structure can be defined as a bond where a hydrogen atom is bonded to a donor atom covalently, which forms weak interactions with a lone pair of electrons of an acceptor atom that is not directly bonded to the group. As mentioned earlier, intra-chain hydrogen bonds are formed within the individual chains of cellulose. The two types of intra-chain hydrogen bonds in cellulose include the O(2)H–O(6) H-bond (formed between the hydroxyl group O(2)H in one ring and an acceptor atom O6 in the next ring) and the O(3)H–O(5) H-bond (formed between the hydroxyl group O(3)H in one ring and an acceptor atom O5 in the next ring). The inter-chain H-bonds include O(2)H–O6 and O(6)H–O3. The main intra-chain hydrogen bond and inter-chain bond are O(3)H–O5 and O(2)H–O6, respectively, and occupy nearly two thirds of the total hydrogen bond network in CNF structure. The remaining one third is occupied by the O(6)H–O2 and O(6)H–O3 intra-chain and inter-chain hydrogen bonds, respectively [35]. These hydrogen bonds (H-bonds) play a vital role in the overall aspects of the cellulose structure. The analysis of the disparity of H-bonds is conducted on H-bonds within the whole CNF structure based on the simulation trajectories. The variations of the H-bonds are investigated in order to understand the contribution of hydrogen bonds to the excellent properties of CNF. Figure 6 shows the overall hydrogen disparity of both the intra-chain and inter-chain H-bonds of Case A in terms of: (a) H-bonds profile with time, and (b) averaged number of H-bonds. It is observed that the total number of H-bonds decreases as the pulling force increases. This decrement can be attributed to the stretching of the bonds leading to some H-bonds exceeding the donor-acceptor cut-off criteria and/or breakage of some H-bonds. We examined both the variations of the intra-chain H-bonds as well as the inter-chain H-bonds. As expected, the intra-chain H-bonds are higher in number than the inter-chain H-bonds, which is expected as defined by theoretical claims. The overall number of intra-chain H-bonds and inter-chain H-bonds were investigated collectively but separately as intra-chain and inter-chain hydrogen bonds for all pull forces: 1000 kJ/mol/nm through 50,000 kJ/mol/nm. The O(2)H–O6 intra-chain bonds were observed to decrease slightly in direct response to the increasing pulling force. The percentage decrement ranges from 0.2% for two independent pulling forces to 1.0% at high pulling force rates (15,000–20,000 kJ/mol/nm). It is of interest to note that extreme sharp decrement in the O(3)H–O5 intra-chain bonds is found ranging from 2.8% at low pulling forces 1000–2500 kJ/mol/nm to 10.1% at 15,000–20,000 kJ/mol/nm pulling force. A distinguishing extreme pulling force of 50,000 kJ/mol/nm reveals significant decrease in H-bonds approximating to 76.7%.

As for the case of inter-chain H-bonds, the overall number of the inter-chain H-bonds is calculated. The disparity of inter-chain H-bonds slightly differed from the trend shown by the intra-chain H-bonds. As the pulling force increased, in some cases there is an indication of slight increment in the inter-chain H-bonds. A general observation on the entire disparity of the H-bond network in the CNF structure shows a decrement of the H-bonds as the pulling force increases, as in Figure 6. 

### 3.4. Donor-Acceptor Distance 

**Figure 7:**
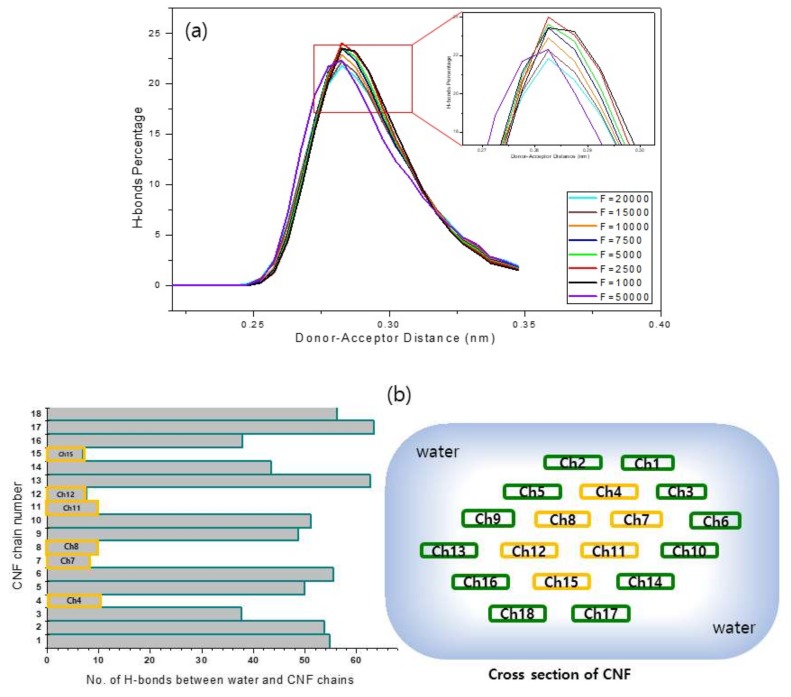
(**a**) Donor-acceptor criterion applied on Case A, and (**b**) H-bonds phenomenon of each chain in with water.

The existence of an H-bond can be qualified using two geometric criteria: r ≤ rHB = 0.35 nm and α ≤ αHB = 30°. We applied these criteria to qualify the existence of strong H-bonds as predicted throughout the simulation. In the case of donor-acceptor criterion, taking into account the O(3)H–O5 and O(2)H–O6 intra-chain H-bonds of cellulose, the donor is oxygen atom which forms a covalent attachment to the hydrogen atom as well-being capable of forming a weak interaction with the lone electron pair of acceptor atom. A donor-acceptor analysis was conducted to qualify the trend. Figure 7a shows the donor-acceptor criterion applied on Case A. The H-bond disparity along with the CNF chains and water is also investigated. Figure 7b shows the result. More H-bonds were prevalent in the chains that were in contact with water. Note that cellulose-water interaction plays a significant role in the crystalline to amorphous transition of cellulose fiber [36]. We calculated the number of hydrogen bonds using the above-mentioned geometric criteria. This means that only the hydrogen bonds that met the above criteria were deemed strong enough to be accepted as a hydrogen bond. If a bond did not range in the given cutoff criteria, then it was disregarded as a weak bond and therefore not included in the count. As observed in Figure 7, at higher pull forces the hydrogen bonds reduced significantly every time the pull force was stepped up. Considering the deviation from the hydrogen disparity at low pull forces and high pull forces, all taking on reference basis of original structure without any pull force, it is agreeable to say that when the cellulose nanofiber is subjected to a pull force, a disruption of the hydrogen bonding occurs, leading to existence of weaker hydrogen bonds within the structure. Applying a cutoff criteria of the donor-acceptor as stated herein returns a count of only the strong hydrogen bonds that meet the criteria. This is a clear indication that subjection of cellulose nanofiber at high pull forces results in a phenomenon of very weak hydrogen bonding within the structure and/or even breakage of the hydrogen bonds altogether.

There are two possible ways of conducting the shear pull (half pull) shear simulation; transverse loading and axial loading. In this study, an axial loading is applied to half of the CNF model. Some atoms of the simulation structure are defined as a dummy group consisting of freeze atoms while applying a pulling force on the unfrozen end. The displacement of the CNF structure along the z-axis when the pulled half chains slide over the other fixed half. As the CNF is sheared, the displacement profile exhibits a zigzag shaped profile. The results indicate a stick-slip separation phenomenon for the CNF chains. It shows that the rate of separation along the pull interface is getting larger as the force is bigger. The number of H-bonds within the CNF structure is investigated. From the initial existing number of H-bonds before the pulling force is applied, this number is shown to be decreased after every pulling force. It is also observed that as the amount of pulling force increases, the number of H-bonds decrement gets higher. 

**Figure 8:**
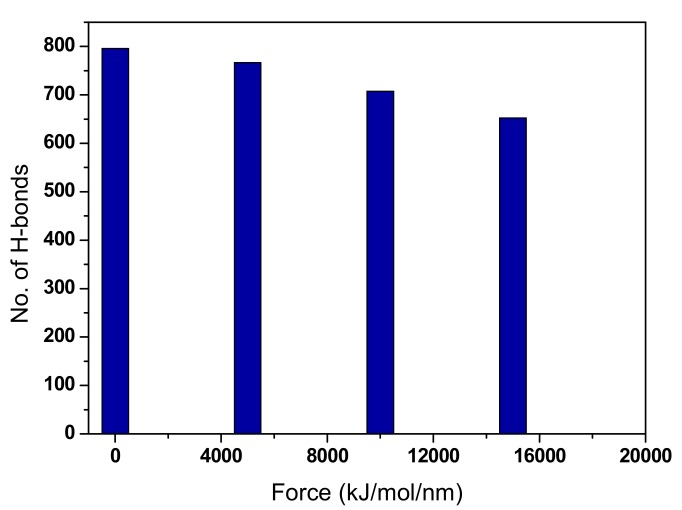
H-bonds during half pull simulation for 200 ps.

Under Visual Molecular Dynamics software as a visualization tool, CNF chains are found to separate along the contact interface depending on the level of pulling force applied. The number of H-bonds is estimated in the whole structure as well as along the normal separation boundary. The number of H-bonds decreases with the increasing pulling force as the CNF structure is pulled away. The rate of decrement is significantly steady without much deviation as shown in Figure 8. Investigating the H-bonds revealed disruption of the hydrogen binding network along the separation interface with a complete separation at high pulling force. Besides H-bond interactions of the CNF chain, it is important to know that Van der Waals interactions also play a crucial role in the stick-slip mechanism of the CNF structure [37]. As described in the methodology, several pull force simulations in Case B are carried out at pulling forces of 5000, 10,000, and 15,000 kJ/mol/nm.

### 3.5. Dihedral Distribution

**Figure 9:**
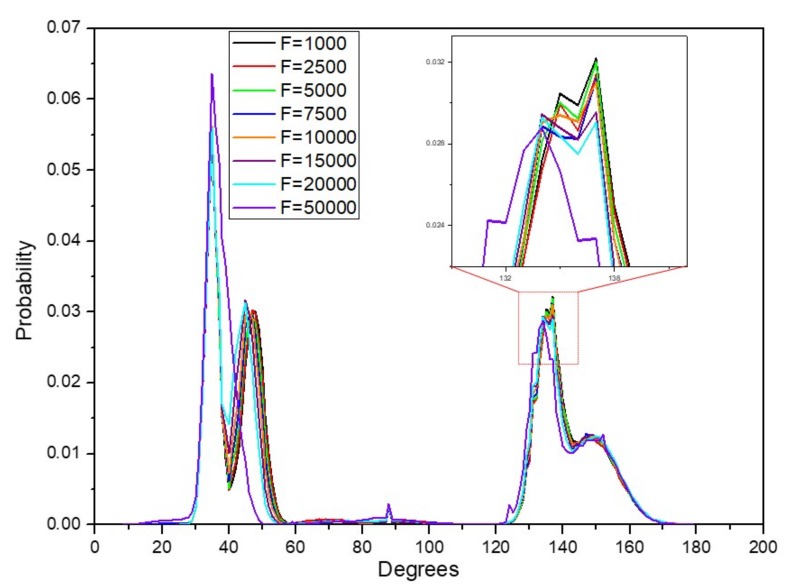
Distribution of dihedral ω.

The conformation of the hydroxymethyl group or the primary alcohol is an important structural feature in cellulose Iβ nanofiber. It adopts different staggered conformations which are often denoted as gt, tg, gg where t and g denote trans and gauche conformation of the dihedral angle ω in relation to the torsion angle formed by O5-C5-C6-O6 and can be denoted as C4-C5-C6-O6 indicating the angle. The conformation of this angle in the cellulose Iβ allomorph is usually at tg conformation which ranges between 120°–180° , mostly at 180° where O5 and C6 are trans to each other. The position of O6–H is paramount in the formation of hydrogen bonding of cellulose chains. We examined the probability distribution of the conformation hydroxymethyl dihedral as represented in Figure 9. We observed two conformations occurring as per our simulation. Deeper examination revealed that the orientation of the hydroxymethyl group of the center chains conformed into the trans-gauche (tg) conformation. Interestingly, the surface chains in contact with water molecules had most of its residue’s hydroxymethyl group conformed into gauche-trans (gt) conformation which usually takes 20°–60°. The angle of the orientation of the dihedral did not present significant deviation. However, it is worth noting that the overall probability of the hydroxymethyl conformation in gt increased as the pull force increased. On the other hand, as pull force increased, the probability of the tg conformation of the dihedral angle decreased. These phenomena are quite similar to cotton cellulose treated with steam at different temperatures [38]. Although this is not related to tensile force, it can be used to elucidate the phenomenon in situations where significant disruption of the hydrogen bonds results in a more disordered structure of cellulose nanofiber.

### 3.6. Mechanical Properties

The pull molecular dynamics simulation of CNF was carried out with the cellulose chains aligned along the z-direction. We applied the periodic pull condition of the GROMACS pull code and conducted the pull force as described in the methodology; Cases A and B. The simulations as per Case A were used for elucidation of strain response to the applied pull force, Young’s modulus, and corresponding effect to the hydrogen bonding. As described in the methodology, different independent pull forces were set from 1000 kJ/mol/nm as a minimum pull force and varied at different simulation cases up to 20,000 kJ/mol/nm. An extreme pull force of 50,000 kJ/mol/nm was carried out to serve as a significantly extreme distinguishing pull force. These magnitudes of pull force contributed to corresponding strains in the range of 0–3.5%. The ratio of the stress applied to the CNF to the corresponding strain were used to plot a stress-strain curve and the linear regression of the resulting stress-strain curve was used to calculate the Young’s modulus of the cellulose nanofiber. The strain of the CNF was obtained in relation to the elongation calculated as the final length of the CNF from the original length before being subjected to the pull force. As mentioned earlier, a group of atoms on one end were frozen (fixed) while the other atoms were subjected to a pull force. The final length of the frozen atoms of the CNF chains was found to be same as its initial length before the MD pull. The remaining portion of the cellulose nanofiber atoms was found to have a significant increment in length in relation to the initial length before the pull MD simulation. This increment was also observed to be consistently increasing each time the pull force was stepped up with the latter increment being higher each time than the former. Cellulose, being a linear polymer subjected to external pull force as in the simulation, we expected that its chains would experience uniform fluctuations in its bond lengths but expected significant jumps occurring in the bond length at the end of the cellulose CNF chains [39]. This anticipated jump was observed in some cases at the end of the chains after the simulation, which was rectified by employing the -nojump option of the gmx trjconv command to check for any atoms that jumped across the box and put them right back. 

**Figure 10:**
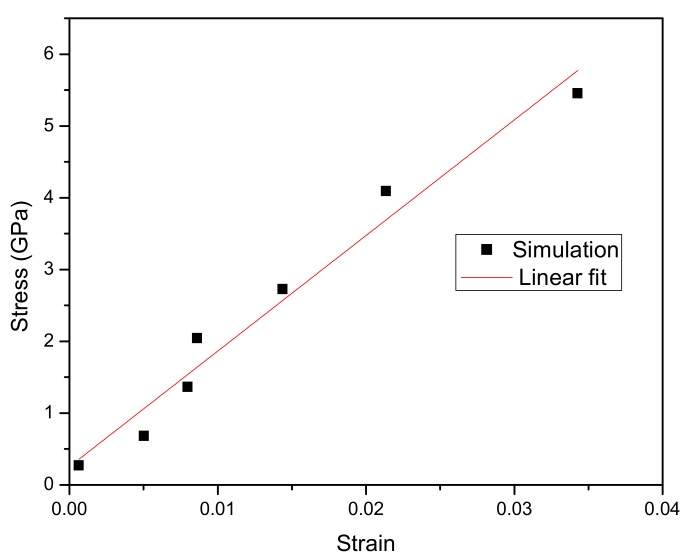
Stress-strain relationship.

The strain values were determined by the ratio of elongation (difference of initial length from the final length) to the initial length of the non-frozen atoms where the pull force was applied with the relation; strain = (l_final_ − l_initial_)/l_initial_. To obtain the corresponding stress to the applied force, we first converted the pull force, kJ/mol/nm into Newton by relation to the Avogadro’s constant per mole then divided it by the cross-sectional area of the CNF simulation structure. While several methods can be used for the estimation of the cross-sectional area, in our case, we employed the simple volume relation; volume = area*length, which yielded the same cross-sectional area as to when estimated from the dimensions of a unit cell multiplied by the number of chains. In this case, we considered the whole length of the CNF in the area calculation. The average end-to-end distance of the simulation structure before and after simulation was obtained. To calculate the volume of the simulation CNF structure, we used Richard’s rolling probe method in a web tool volume assessor program known as 3Vee (Voss Volume Voxelator) to calculate the volume of our CNF molecule by rolling a virtual probe on the surface of our molecule file loaded using the key structure file that formed the basis of our entire simulation. We selected the radius of the virtual probe as 6 Å (0.6 nm) and a grid resolution of 0.75 Å voxels (0.075 nm voxels) was used [40]. Figure 10 shows the stress vs. strain results for the individual simulations and its slope of the linear fit represents the Young’s modulus of CNF.

In molecular mechanics of a polymer, linear polymer in particular, its stress response is closely correlated with its structural properties such as mean-square end-to-end length, bond length, radius of gyration of the chain distributions, and dihedral angle. Just like any other dense linear polymer, it is expected that under any subjection of constant strain rate (constant pull force), the chains of the CNF would begin to deform by stretching of the bonds [39]. In our study, we focused on structural properties such as end-to-end length of the chains as a function of simulation time of the constant strain rate loading produced by the applied constant tensile pull force on the CNF.

We investigated the end-to-end length behavior as well. We observed that the end-to-end length grows linearly with strain. The stress-strain relationship deduced a Young’s modulus of 161 GPa for all the 18 chains of CNF. This value of modulus obtained using the modified AA-OPLS force field for carbohydrates was a bit higher as compared to the previously achieved values using previous versions of force field. A study conducted to investigate mechanical properties of cellulose nanofibers by MD simulations reported a Young’s modulus of 157 GPa [18]. This study employed OPLS force field and the method in which the pull force was applied differed greatly from our methodology. Another research group [21] conducted an MD simulation to investigate tensile strength of crystalline cellulose Iβ by subjecting the simulation box to tensile deformation strains using LAMMPS (Large-scale Atomic/Molecular Massively Parallel Simulator). The resulting modulus ranged between 107.8 GPa to 113.5 GPa in the z-axis alignment in the simulation box depending on the value of the strain rate used. As it has been reported before, results of molecular dynamic simulation of CNF are highly dependent on the force field used to carry out the simulations. Different force fields have been reported to yield variations in the simulated Young’s modulus. The calculated modulus also depends on the number of chains in the simulation model which varied among the groups as well. More efforts towards molecular dynamics simulations need to be upheld for proper stabilization of the force field deviations.

## 4. Conclusions

CNF fibers and films are currently being applied in many application disciplines. It is important to understand that the mechanical performance of the CNF-derived materials is dependent on the Young’s modulus and the underlying interlayer shear modulus. The elastic modulus of cellulose Iβ was studied by atomistic simulation. A Young’s modulus deduced from the stress-strain relationship was found to be 161 GPa for all the 18 chains of the model. The population of hydrogen bonds within the crystalline cellulose structure was found to decrease as the amount of pulling force increased. This study gives an insight into how hydrogen bonding changes as a function of deformation in the axial direction of crystalline cellulose. The elusive behavior of cellulose hydrogen bonds gives helpful insight when using CNF composites or devices designed for applications where tensile forces would be applicable. Other interactions such as coulombic forces and Van der Waals forces are key factors whose role could be significant in the strength of cellulose crystals, but these interactions were beyond our scope. The overall hydroxymethyl conformations of the crystalline cellulose structure taking the tg conformation was found to be dependent on the amount of pull force. As the pull force increased, the number of tg conformations reduced which may have translated to the observed decrease in hydrogen bonds. A separation mechanism was also observed when some chains were pulled. This phenomenon showed a severe decrement of the H-bonds as well.
